# The impact of single positive surgical margin features on biochemical recurrence after robotic radical prostatectomy

**DOI:** 10.1590/S1677-5538.IBJU.2017.0702

**Published:** 2019

**Authors:** Ismail Evren, Ahmet Hacıislamoğlu, Mithat Ekşi, Abdullah Hızır Yavuzsan, Fırat Baytekin, Yunus Çolakoğlu, Didem Canoğlu, Volkan Tugcu

**Affiliations:** 1 Department of Urology, Bakirkoy Dr. Sadi Konuk Training and Research Hospital, Istanbul, Turkey;; 2 Department of Pathology, Bakirkoy Dr. Sadi Konuk Training and Research Hospital, Istanbul, Turkey

**Keywords:** Margins of Excision, Prostatectomy, Pathology

## Abstract

**Objective:**

Parameters predictive of biochemical or clinical recurrence after Radical Prostatectomy (RP) were determined as pre-treatment PSA value, pathologic tumor stage, tumor grade and presence of Positive Surgical Margin (PSM), extracapsular extension and seminal vesicle invasion and the status of pelvic lymph nodes. The aim of our study is to evaluate the effect of additional features in patients undergoing RP in our clinic.

**Materials and Methods:**

We studied 556 RP operations performed between 2009 and 2016 for prostate cancer at this clinic. Preoperative and postoperative data of the patients were retrospectively reviewed. RP specimens were examined by two pathologists specialized in this subject. Of these patients, 78 (14.02%) patients with PSM were included in the study. The pathology slides of these patients were reassessed. The length of PSM (mm), localization (apex, basis and posterolateral) and Gleason pattern at this margin was determined and statistical correlations with BCR were calculated.

**Results:**

The mean follow-up after the RP of 41 patients included in the study was 37.4 ± 13.2 months. During the follow-up period of the patients, BCR was observed in 16 patients (39.02%). No statistically significant difference was observed in age and prostate volume between the groups with and without BCR development (p > 0.05). Preoperative PSA level was found to be statistically significantly higher in the group with BCR development compared to the group without recurrence (p = 0.004). In-group comparisons in each aforementioned Gleason score groups were performed in terms of BCR development and the preoperative Gleason score in the group with development of recurrence was found to be statistically significantly higher compared to the group without recurrence (p = 0.007). The length of the surgical margin was measured as 7.4 ± 4.4 mm in the BCR-developing group and 4.7 ± 3.8 mm in the no-BCR- developing group; it was statistically significantly higher in the group with development of recurrence (p = 0.03).

**Conclusion:**

Length and location of the PSM and the Gleason score detected in the PSM region could not predict biochemical recurrence according to the results of this present study. However high preoperative PSA value is an independent prognostic factor for biochemical recurrence.

## INTRODUCTION

Radical prostatectomy (RP) is the standard of care for young men who have organ confined prostate cancer (1). In the pathological examination of the specimen, the presence of cancer cells in the resected area stained with ink is called positive surgical margin (PSM) and is considered as incomplete resection. In general, PSM ratios are reported between 6 – 41% after RP (2, 3).

PSM is regarded as an independent risk factor for the development of biochemical recurrence (BCR), local recurrence and distant metastasis (4).

In this study, we aimed to determine whether the features of single positive surgical margins have any impact on biochemical recurrence in patients who underwent robot assisted radical prostatectomy (RARP).

## MATERIAL AND METHODS

We studied 556 RARP operations performed between 2009 and 2016 for prostate cancer at our clinic. Preoperative and postoperative data of the patients were retrospectively reviewed. The surgeries were performed in the Vinci SI or XI surgical systems® (Intuitive Surgical Inc., Sunnyvale, California, USA). All RARPs have been performed by a single surgeon (V.T) using the Frankfurt technique described by Wolfram and colleagues (5).

Age (year), prostate volume measured by transrectal ultrasonography (cc), preoperative PSA level (ng / mL), Gleason score of the preoperative biopsy, Gleason scores of the RP specimen, extraprostatic extension (EPE), PSM and postoperative PSA level of the patients were recorded. The BCR criterion (biochemical recurrence) was accepted as PSA level above 0.2 ng / mL in 2 consecutive measurements. Of these patients, 78 (14.02%) patients with PSM and that did not require or accept any adjuvan therapies after radical prostatectomy were included in the study. The pathology slides of these patients were reassessed. The length of PSM (mm), localization (apex, basis and posterolateral) and Gleason pattern at this margin was determined and statistical correlations with BCR were calculated. Patients with seminal vesicle involvement in RP specimen (n = 6, 7.6%) and PSM in multiple foci (n = 22, 28.2%) were excluded in order to provide a more explicit and clearer assessment of the effect of these PSM factors on BCR. Nine (11.5%) patients who had no regular PSA follow-up were also excluded from the study. Finally, a total of 41 patients (52.5%) were included in the study.

Patients were divided into two groups as with BCR (n = 16, 39%) and without BCR (n = 25, 61%) groups. The data obtained from these groups were statistically compared.

### Pathological Evaluation

RP specimens were examined by two pathologists specialized in this subject. All of the prostatic tissue was processed into 4 – 5 mm thick cross - sections from the apex through the bladder neck. The entire surface was marked as surgical border with India ink. Microscopic examination of the neoplastic areas in contact with the ink was evaluated as surgical margin positivity. Positive surgical margin length was measured with a ocular micrometer in the microscope. Incidental incisions through the tumoral tissue were not evaluated as positive surgical margin. Surgical border evaluation in EPE areas was done as described.

### Statistical Methods

Mean, standard deviation, median, lowest, highest, frequency and ratio was used for the descriptive statistics of the data. The distribution of the variables was analyzed with the Kolmogorov - Smirnov test. Independent sample t test was used for the analysis of quantitative independent parametric data and Mann - Whitney U Test was used for the analysis of the nonparametric data. The independent qualitative data were analyzed by Chi - square test and the Fisher test was used when Chi - square test conditions were not met SPSS 22.0 program was used to perform the statistical analyses.

## RESULTS

Mean age, preoperative PSA level, and prostate volume were 61.7 ± 5.1 years, 10.7 ± 7.2 ng / mL and 39.1 ± 19 cc, respectively. In terms of preoperative Gleason scores, Gleason 3 + 3 = 6 was detected in 19 patients (46.3%), Gleason 3 + 4 = 7 and 4 + 3 = 7 were detected in 10 patients (24.4%), and Gleason 4 + 4 = 8 was detected in 2 patients (4.9%). Gleason scores obtained after the pathological evaluation of RP specimen were as follows; Gleason 3 + 3 = 6 in 11 patients (26.8%), Gleason 3 + 4 = 7 in 15 patients (36.6%), Gleason 4 + 3 = 7 in 10 patients (24.4%), Gleason 4 + 4 = 8 in 4 patients (9.8%) and Gleason 4 + 5 = 9 in 1 patient (2.4%). From Gleason scores of TRUS Bx to prostatectomy specimens, upgrading and downgrading was detected in 31.7% and 4.8% of the patients, respectively. The mean length of the surgical margin in non recurrence and in recurrence groups were 4.7 ± 3.8 and 7.4 ± 4.4 mm, respectively. In non recurrence group, Gleason pattern at the PSM area was 3 for 88% of the patients (n = 22) and for 12% was 4 (n = 3). In the recurrence group, Gleason pattern at the PSM area was 3 for 62.5% of the patients (n = 10) and for 37.5% was 4 (n = 6). The mean Gleason scores at the PSM area in non recurrence and recurrence groups were 3.1 ± 0.3 and 3.4 ± 0.5, respectively. PSM was detected to be at the posterolateral prostate in 25 (61.0%) patients and at the prostatic apex in 16 (39.0%) patients. Review of the RP specimen revealed absence of EPE in 23 patients (56.1%) and presence of EPE in 18 patients (43.9%). There was not any PSM at the EPE area for any patients. These data are presented at Table-1.

**Table 1:** Preoperative and postoperative parameters.

		Min-Max			Median	Mean ± SD / n/ %		
Age (years)		45	–	71	62	61.7	±	5.1
Preoperative PSA (ng/mL)		1.8	–	30	9.7	10.7	±	7.2
Prostate volume (cc)		17	–	96	32	39.1	±	19
**Gleason Score**								
Preoperative	3+3					19		46.3%
	3+4					10		24.4%
	4+3					10		24.4%
	4+4					2		4.9%
RP Specimen	3+3					11		26.8%
	3+4					15		36.6%
	4+3					10		24.4%
	4+4					4		9.8%
	4+5					1		2.4%
**Surgical Margin**								
Length (mm)		1	–	18	5	5.7	±	4.2
Gleason Patern		3	–	4	3	3.2	±	0.4
Location	Posterolateral					25		61%
	Apex					16		39%
EPE	(-)					23		56.1%
	(+)					18		43.9%

The mean follow-up after the RP of 41 patients included in the study was 37.4 ± 13.2 months. None of the patients died during follow-up period.

During the follow-up period of the patients, BCR was observed in 16 patients (39.02%). No significant difference was observed in age and prostate volume between the groups with and without BCR development (p > 0.05). Preoperative PSA level was found to be significantly higher in the group with BCR development compared to the group without recurrence (p = 0.004). In - group comparisons in each aforementioned Gleason score groups, BCR development and the preoperative Gleason score in the group with recurrence was found to be significantly higher compared to the group without recurrence (p = 0.007). When the RP specimen Gleason scores were compared, no significant difference was found between the two groups (p > 0.05). There was no significant difference in EPE presence between the groups (p > 0.05) (Table-2).

**Table 2:** Recurrence and relationship with parameters.

		Recurrence (-)				Recurrence (+)				P	
		Mean ± SD / n/ %			Median	Mean ± SD/ n/ %			Median		
Age (years)		61.5	±	5.7	62	62.1	±	4.1	61.5	0.745	^t^
Preoperative PSA (ng/mL)		8.3	±	5.6	6.7	14.4	±	8	12.2	**0.004**	^m^
Prostate volume (cc)		38.6	±	19.1	32	40	±	19.3	32	0.611	^m^
**Gleason Score**											
Preoperative	3+3	15		60%		4		25%		**0.007**	^X²^
	3+4	7		28%		3		18.8%			
	4+3	2		8%		8		50%			
	4+4	1		4%		1		6.3%			
RP Specimen	3+3	8		32%		3		18.8%		0.591	^X²^
	3+4	11		44%		4		25%			
	4+3	4		16%		6		37.5%			
	4+4	2		8%		2		12.5%			
	4+5	0		0%		1		6.3%			
**Surgical Margin**											
Length (mm)		4.7	±	3.8	3.5	7.4	±	4.4	8	**0.030**	^m^
Gleason Pattern		3.1	±	0.3	3	3.4	±	0.5	3	0.057	^m^
Location	Posterolateral	18		72%		7		43.8%		0.070	^X²^
	Apex	7		28%		9		56.3%			
EPE	(-)	16		64%		7		43.8%		0.202	^X²^
	(+)	9		36%		9		56.3%			

^t^ = t test;^m^ = Mann-whitney u test; ^X²^ = Chi-square test; **RP** = Radical prostatectomy; **EPE** = Extraprostatic extension.

**Figure 1A and 1B:**
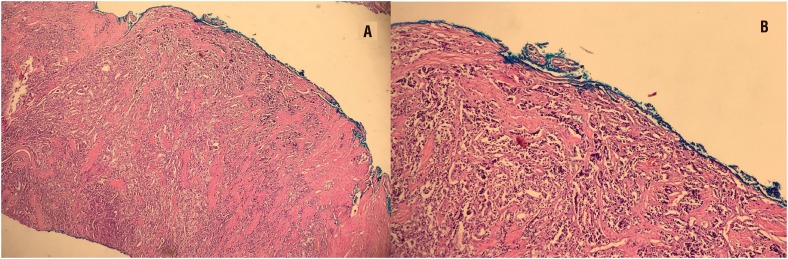
Acinar adenocarcinoma with Gleason 4 pattern. It is shown with 40 X and 100 X magnification respectively. Positive surgical margin length 3 mm.

The mean length of the PSM was significantly higher (p = 0.03) in the BCR group (7.4 ± 4.4 mm) than in the non - BCR group (4.7 ± 3.8 mm). No significant differences were found between the two groups in the Gleason pattern in the PSM region and the localization of the PSM among other factors of surgical margin (p > 0.05) (Table-2). Image of the surgical margin in shown in Figures 1A and 1B.

**Figure 2:**
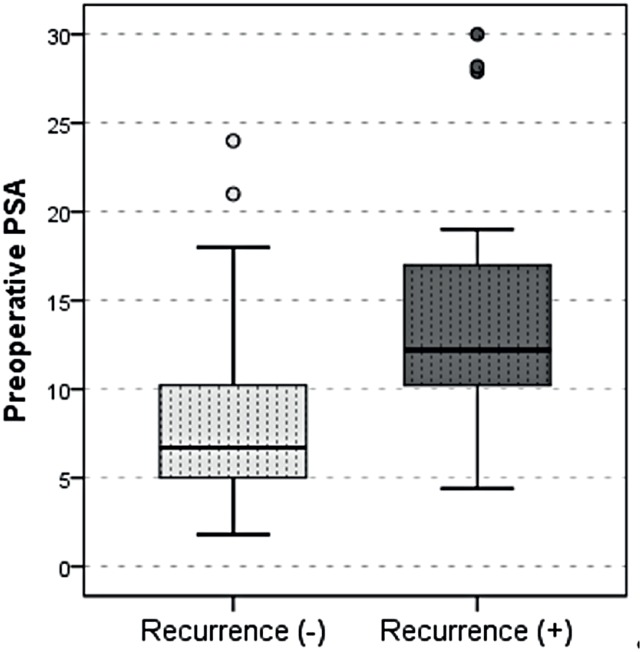
Preoperative PSA levels and association with biochemical recurrence.

When all the data obtained by logistic regression model were evaluated in terms of biochemical recurrence, preoperative PSA, preoperative Gleason score and length were found to be significantly efficient in the prediction of the patients with and without recurrence in the univariate model (p ˂ 0.05). On the other hand, as a result of the multivariate reduced model, preoperative PSA was found to be a significant and independent predictor of the patients with and without recurrence (p = 0.019) (Tables 3 and Figure 2).

**Table 3:** Parameters predicting biochemical recurrence.

	Univariate Model					Multivariate Model				
	OR	% 95 CI			P	OR	% 95 CI			P
Age (years)	1.02	0.9	–	1.16	0.738					
Preoperative PSA (ng/mL)	1.15	1.02	–	1.28	0.019	1.15	1.02	–	1.28	0.019
Preop Gleason S.	2.71	1.25	–	5.86	0.011					
Specimen Gleason S.	1.85	0.95	–	3.60	0.069					
Prostate weight (cc)	1	0.97	–	1.04	0.816					
EPE	2.29	0.63	–	8.23	0.206					
PSM Length (mm)	1.18	1	–	1.41	0.042					
Gleason Pattern at PSM	4.40	0.91	–	21.25	0.065					
PSM Location	3.31	0.88	–	12.35	0.075					

Logistic Regression Model

**EPE =** Extraprostatic extension; **PSM =** Positive surgical margin

## DISCUSSION

RP is the standard treatment for localized prostate cancer in young patients (1). Parameters predictive of biochemical or clinical recurrence after RP were determined as pre - treatment PSA value, pathologic tumor stage, tumor grade and presence of PSM, extracapsular extension and seminal vesicle invasion and the status of pelvic lymph nodes (4, 6-8). The preoperative PSA values of the study group with development of BCR included in this present study were found to be significantly higher in both univariate and multivariate analyzes compared to the group without development of BCR. In addition, patients with a high Gleason score in biopsy specimen were found to have significantly higher BCR rates, but this parameter was not an indepent predictor by multivariate analysis.

PSM may develop as a result of incision of a tumor with extracapsular invasion or through an inappropriate incision performed in organ - confined cancer (9). The variable rates of EPE (6 – 41%) are in parallel to surgical technique improvements and with an increase in RP indication for organ - confined disease, which relates to wider PSA screening in the last two decades (2, 3). Tewari et al. reported the rates of PSM after open, laparoscopic and robotic radical prostatectomy (ORP, LRP, RARP) as 24.2%, 20.4% and 16.2%, respectively (10). In addition, PSM ratio in pT2 and pT3 disease have been reported as 18% and 40%, respectively (11).

PSM has been shown to be associated with prognostic parameters such as BCR, local recurrence, and distant metastasis (4, 11-13). However, since not every patient with PSM develops BCR, it is critical to determine which patient needs adjuvant treatment after RP and which of the adjuvant treatments presented in the condition of PSM (such as adjuvant radiotherapy) is an overtreatment.

Five - year BCR rate has been reported as 42 – 64% in patients with PSM (14). Epstein et al. compared patient groups with PSM and negative surgical margins (NSM) and reported the progression - free survival as 55% and 79%, respectively (15). Multivariate analysis revealed that PSM was an independent risk factor for 10 - year progression - free survival in a study performed in 1389 patients (16). This association between PSM and BCR has been demonstrated only in the group with a high risk prostate cancer, while it could not be demonstrated in the lower risk groups (3, 17, 18).

The results of the studies on the length of the PSM are controversial. Fromont et al. reported that the length of the PSM in the posterolateral area following LRP could be a major risk factor for local recurrence and the rate of residual tumor was around 60% in the presence of a PSM longer than 5 mm (19). The risk of BCR was demonstrated to be significantly increased in patients with multifocal and > 3 mm PSM in a study performed in 7160 patients with 32 years of follow-up (20). Shikanov et al. reported similar BCR rates in patients with PSM < 1 mm and in patients with NSM, while the patients with a PSM > 3 mm had the lowest chance for a biochemical recurrence - free survival (21). In this present study, the length of PSM was significantly higher in the group with BCR although this difference could not be demonstrated by multivariate analysis. By the ROC analysis, the cut - off value of length of the PSM for BCR recurrence was found “7.5 mm” in our study which provide a 88% specifity and 75% positive predictive value (p = 0.023).

The effect of presence of PSM in one or more regions is controversial in the literature. Presence of PSM in multiple foci has been reported to create significant differences in terms of disease recurrence and need for a secondary treatment compared to the presence of PSM in a single focus (22, 23). Vis et al., on the other hand, reported no significant difference between multiple PSM and solitary PSM by both univariate and multivariate analysis (11). Patients with only a single focus of PSM were included in this present study to provide the homogeneity of the data in this present study.

No consensus has been reached on the most frequent region of PSM after ORP, LRP and RARP and which region(s) are associated with BCR. The most common region of PSM after RARP has been reported to be prostatic apex region in some studies (24-26). In contrast, posterior or posterolateral region has also been reported to be the most frequent area of PSM after RARP in some other publications (27, 28). Some groups suggested that BCR was independent of location (7, 29-31). PSM located in the posterolateral region has been found to be associated with a poorer prognosis in some studies (4, 32). In a study performed with 2234 patients, PSM was reported to be associated with 5 - year clinical and BCR when it was located only in the base region, while it was found to have no effect on BCR when it was located in other anatomic regions or it was multiple, as long as it was base negative (22, 33). Epstein et al. reported that solitary apical PSM was associated with a higher recurrence rate and a shorter time to progression (15); however, no such finding could be demonstrated in other publications (7, 13, 30, 31). Fesseha et al., in their study with 590 patients, stated that the rate of BCR was similar in two groups of patients, one with presence of apical PSM and the other with NSM (30). The most frequent location of PSM found in this present study was posterolateral region in 61% and the remaining 39% were located in the apex. Univariate and multivariate analysis of the data of this present study revealed that BCR was independent of the location.

The effects of presence of focal PSM and EPE on BCR - free survival were analyzed in a study and two groups were compared and development of BCR was reported to occur in 92% and 58% respectively in the two groups (34). Paulson et al. found the 10 - year disease progression rate to be 70% in the presence of EPE and PSM (35). Lowe and Lieberman, in their study, in which they compared pT3 disease and organ confined disease, found a significantly higher rate of disease progression in pT3 disease (23). In this study, no significant effect of the presence of EPE on increased rate of BCR was demonstrated on this subset of patients with positive surgical margins.

Gleason score determined at a PSM area was reported to be an independent risk factor for BCR in some studies (36-38). Savdie et al. reported that the risk of PSA recurrence doubled in patients with Gleason 4 and 5 patterns at a PSM and the risk was similar with the NSM patient group among patients with a Gleason 3 score at a PSM (38). In this present study, no significant difference was found in the mean Gleason scores determined at the PSM areas between the groups with and without recurrence similar with some other study in the literature (39). Also, there was no significant difference between the groups with and without recurrence according to a Gleason 3 or Gleason 4 pattern at the PSM area (chi - square and Fisher - exact test). None of the patients had Gleason 5 pattern at the PSM area in our study.

The main limitations of this study were the retrospective structure of the study, short follow-up time and the small number of patients. In relation to positive surgical margins after radical prostatectomy, most of the studies were before the Gleason updates in 2004, therefore prospective studies with larger number of patients are required in order to define the patient groups with the specifications of the surgical margin that would benefit most from the adjuvant radiotherapy after RP.

## CONCLUSIONS

Location of the single PSM and the Gleason score detected at the PSM area could not predict biochemical recurrence after robotic radical prostatectomy. However, the length of PSM has positive predictive value about BCR. Preoperative PSA value is an independent prognostic factor for biochemical recurrence.
